# Should kissing balloon inflation after main vessel stenting be routine in the one-stent approach? A systematic review and meta-analysis of randomized trials

**DOI:** 10.1371/journal.pone.0197580

**Published:** 2018-06-27

**Authors:** Ming Zhong, Biao Tang, Qiang Zhao, Jian Cheng, Qiangsong Jin, Shenwen Fu

**Affiliations:** Department of Cardiology, Jinhua Municipal General Hospital, Jinhua, Zhejiang, China; University of Bologna, ITALY

## Abstract

The KBI (kissing balloon inflation) technique is considered the default strategy for the two-stent approach in real world practice. Studies comparing KBI and No-KBI in patients undergoing the one-stent approach have reported conflicting results. The meta-analysis was performed to compare the clinical outcomes of the KBI strategy and the No-KBI strategy for coronary bifurcation lesions in the one-stent approach. Five randomized studies were included, and a total of 1264 patients were involved in the meta-analysis. The primary outcome was cardiac death. The secondary end points were stent thrombosis, MI (myocardial infarction), target lesion revascularization (TLR), target vessel revascularization (TVR), and main vessel and side branch restenosis. Compared with the No-KBI strategy, the KBI strategy was associated with a significant reduction in side branch restenosis (OR: 0.44, 95% CI: 0.30–0.64, p<0.001). A high risk of main vessel restenosis was found in the KBI group (OR: 2.96, 95% CI: 1.74–5.01, p<0.001). There were no significant differences in rates of cardiac death (OR: 1.89, 95% CI: 0.60–5.95, p = 0.28), stent thrombosis (OR: 0.98, 95% CI: 0.19–4.94, p = 0.98), MI (OR: 0.68, 95% CI: 0.33–1.44, p = 0.30), TLR (OR 1.14, 95% CI 0.68–1.90, p = 0.62), or TVR (OR 1.27, 95% CI 0.75–2.16, p = 0.38). Compared with the No-KBI strategy, the KBI strategy reduced the incidence of side branch restenosis and increased the risk of main branch restenosis in the one-stent approach. However, the clinical outcomes were similar between the KBI and No-KBI groups.

## Introduction

Coronary bifurcation lesion is regarded as one of the most challenging lesions and is known to be associated with lower angiographic success rates, higher risk of procedural complications, and higher restenosis rates than non-bifurcation lesions[1].

KBI (kissing balloon inflation) is the standard strategy in bifurcation lesions that are treated with the two-stent technique[2–4], Unfortunately, the benefit of KBI in the one-stent approach remains uncertain due to poor clinical data[5,6]. Several recent retrospective trials that compared the KBI strategy with the No-KBI strategy in patients undergoing the one-stent technique did not show any detectable advantages in the clinical outcome [7–9].

Therefore, the purpose of the present study is to answer whether or not KBI should routinely be performed after main vessel stenting in the one-stent approach.

## Materials and methods

### Search strategy and study selection

Literature comparing the KBI and the No-KBI strategy after main vessel stenting for coronary bifurcation lesions was acquired through searching Medline, Pubmed, Embase, the Cochrane Controlled Trials Registry and Clinical Trials Registry from January 2007 to August 2017. We used search terms such as bifurcation, coronary bifurcation, kissing, randomized, and clinical trial. References from reviews and selected articles were further screened. The inclusion criteria were as follows: (1) RCTs (randomized controlled trials) between routine kissing balloon versus provisional technique, (2) English-language study, and (3) clinical outcomes were reported, and follow-up time was at least 6 months. The exclusion criteria were as follows: (1) non-randomized or non-English-language studies, and (2) studies with duplicate publication or different follow-up periods from the same sample origin.

### Data extraction and quality assessment

All relevant articles were independently reviewed by two investigators (M.Z. and B.T.) to assess the eligibility of each article and abstract with the standardized data abstraction forms, and any disagreement was resolved by a third investigator (J.C.). The following data were extracted from the eligible RCTs: study name, publication date, first author, baseline demographics, procedural characteristics, clinical outcomes, and angiographic results at follow-up. The methodological qualities of the included randomized studies were assessed using the Jadad scale.

### Statistical analysis

Dichotomous variables are expressed as odds ratios (OR) and were calculated using the Mantel-Haenszel method and 95% confidence intervals (CI). The heterogeneity among trials was evaluated with Cochran’s Q test and I^2^ statistic, and high heterogeneity was considered present for p<0.10 of the Q test and/or I^2^ ≥50%. The random effects model was used if there was significant heterogeneity across trials. If not, the fixed effects model with the Mantel-Haenszel method was performed. Egger’s test was employed to test for funnel plot asymmetry at the p<0.05 level of significance. The influence of a single study on the summary estimates was examined graphically by checking how the elimination of each study affected the results. Meta-regression was applied to further explore heterogeneity. The p-value threshold for significance was 0.05. Statistical analyses were conducted using STATA software 12.0 (StataCorp, College Station, TX, USA).

## Results

### Search results, baseline characteristics

**Fig 1:**
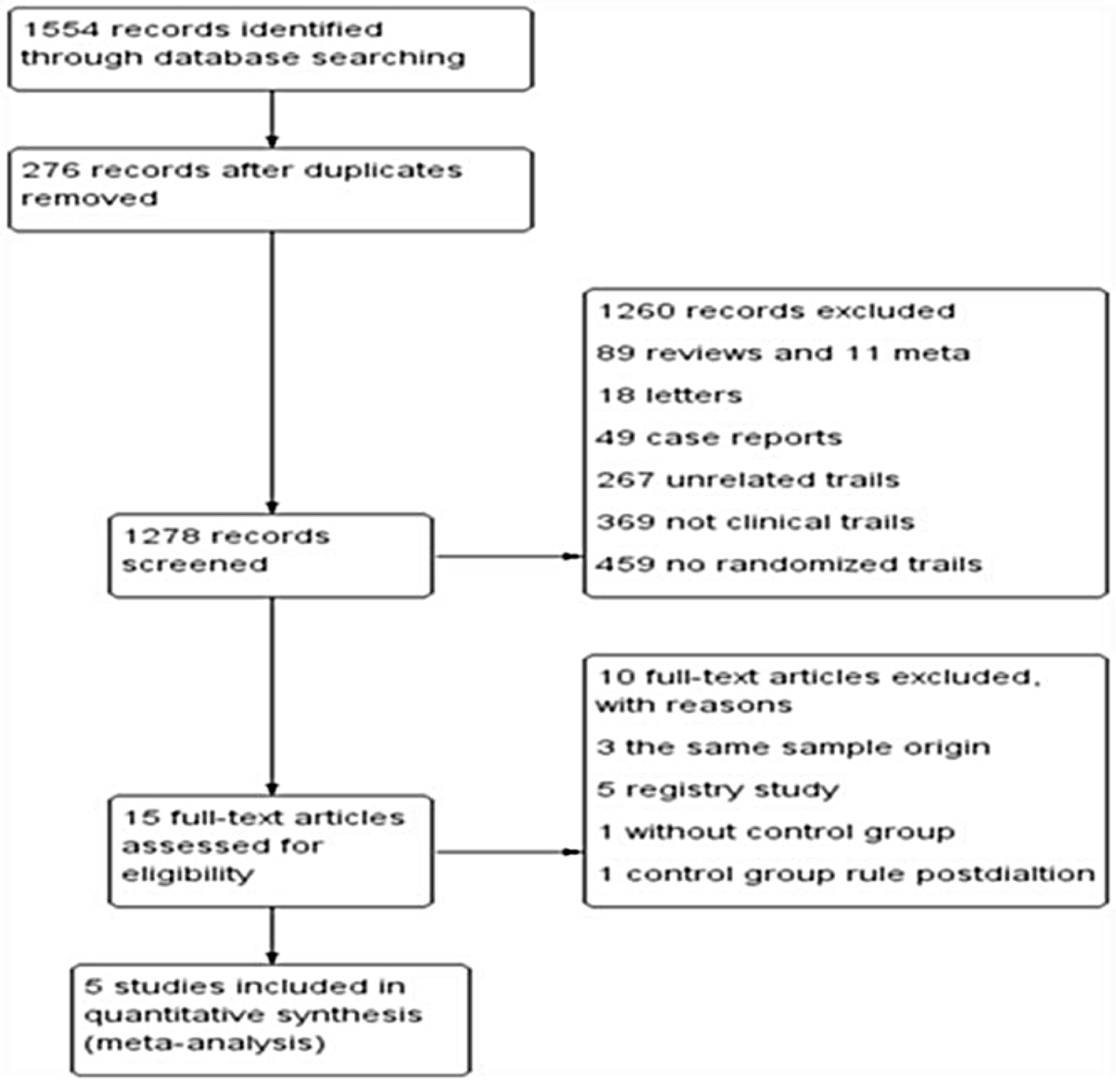
Study selection flow diagram. Summary of progress through the stages of search and eligible study identification.

Five eligible studies[10–14], including a total of 1264 patients, were identified in the present meta-analysis, with 631 patients in the KBI strategy group and 633 in the No-KBI strategy group **(Fig 1)**. The quality of each study was high on the Jadad scale. The baseline, procedural and follow-up characteristics are listed in **Table 1** and **Table 2**. The new-generation drug-eluting stents were used across the studies. The kind of stents for each study is listed in **Table 3**. The kissing balloon inflation technique routine was used in the KBI group after main vessel stenting in all studies. All studies in the No-KBI group performed the kissing balloon technique only if the thrombolysis in myocardial infarction (TIMI) flow grade was <3 in the side branch after main vessel stenting, except for the Nordic III and the Cross trial, in which the stent was used directly. Clinical follow-up periods for the five trials ranged from 6 to 36 months. One trial reported outcomes beyond one year[12]. Angiographic follow-up and quantitative coronary angiography were performed 6–9 months after the index procedure. The rate of angiographic follow-up was 83%. Assessment of the treated vessels with intravascular ultrasound after stenting and at follow-up was performed in 3 of the studies [11–12,14]. Bifurcation lesions that included the left main stem were reported in 150 cases in 2 of the studies [10,14].

**Table 1:** Baseline characteristics.

Study	Year	Patients	Age	Man	DM	HP	LVEF	Jadad
Yamawaki et al.	2017	113	69	90	41	82	64.3 ± 10.0; 63.7 ± 9.83	4
Nordic III	2011	477	65	347	81	303	58.0±11.0; 59.0±10.0	5
CROSS	2015	306	61	211	91	175	60.9±7.0; 62.2±5.7	4
SMART-STRATEGY	2012	258	62	213	70	145	59.3±10.7; 60.5±.3.0	4
THUEBIS	2009	110	66	82	28	91	62.4±9.9; 60.1±9.5	5

Values are the mean±SD; DM = diabetes mellitus; HP = hypertension; LVEF = left ventricular ejection fraction.

**Table 2:** Procedural and follow-up characteristics.

Study	KBI	NO-KBI	LM	Dual antiplatelet(months)	IVUS	MACE	Follow-Up(months)
Yamawaki et al.	56	57	No	NA	Yes	cardiac death, TLR, MI	36
Nordic III	238	239	Yes	12	No	cardiac death, MI, stent thrombosis, TLR	6
CROSS	151	155	No	12	Yes	comprising death, MI, TVR, TLR, MI	12
SMART-STRATEGY	130	128	Yes	NA	Yes	cardiac death, MI, TVR	12
THUEBIS	56	54	No	6	No	cardiac death, TLR, stent thrombosis.	6

KBI = kissing balloon inflation; LM = left main; IVUS = intravascular ultrasound; MACE = major adverse cardiac events; TLR = target lesion revascularization; MI = myocardial infarction

TVR = target vessel revascularization; NA = not available.

**Table 3:** Kind of stent for each study.

Study	Yamawaki et al.	Nordic III	CROSS	SMART-STRATEGY	THUEBIS
KBI (%)	Everolimus-eluting stents	Sirolimus-eluting cypher select+	Sirolimus-eluting stents 47 (31.1); Paclitaxel-eluting stents 17 (11.3); Everolimus-eluting stents 33 (21.9); Zotarolimus-eluting stents 44 (29.1); Others 10 (6.6).	Sirolimus-eluting stent 60 (46.9); Everolimus-eluting stent 40 (31.3); Other drug-eluting stents 28 (21.9).	Taxus Express or Taxus Liberte stents
NO-KBI(%)			Sirolimus-eluting stents 36 (23.2); Paclitaxel-eluting stents 21 (13.5); Everolimus-eluting stents 36 (23.2); Zotarolimus-eluting stents 53 (34.2); Others 9 (5.8).	Sirolimus-eluting stent 62 (47.7); Everolimus-eluting stent 35 (26.9); Other drug-eluting stents 33 (25.4).	

KBI = kissing balloon inflation.

## Quantitative data analysis

### Cardiac death

**Fig 2:**
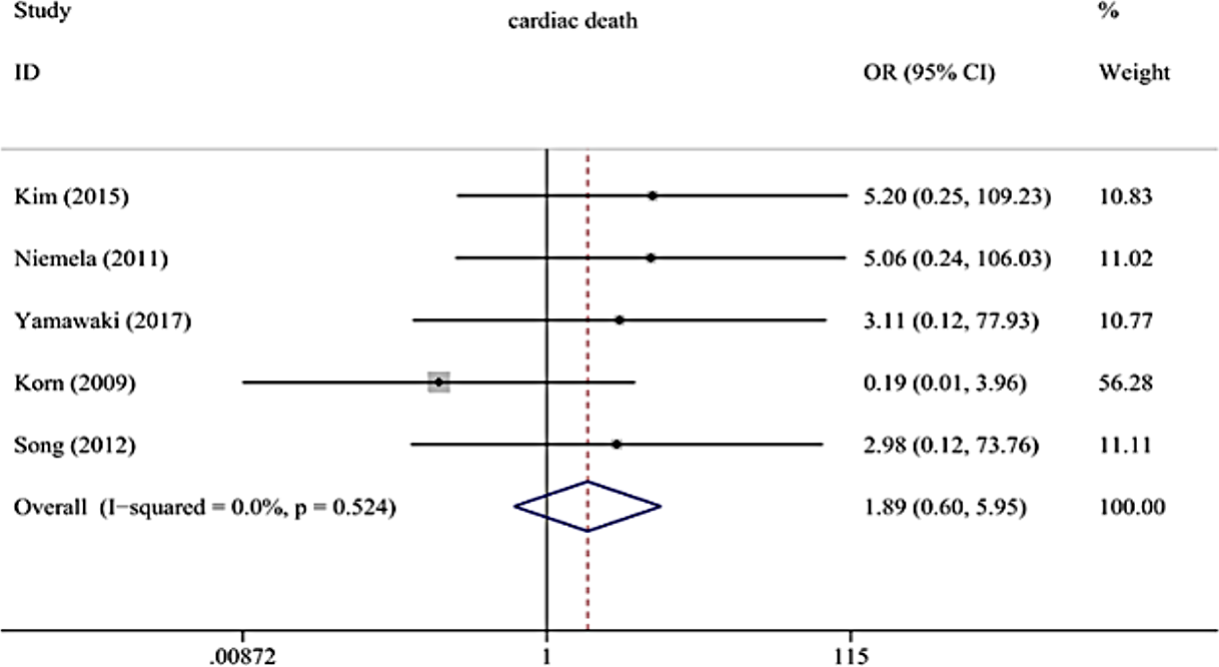
Forest plot of primary outcomes; cardiac death.

As shown in (**Fig 2)**, the KBI strategy had a similar risk of cardiac death, compared with the No-KBI strategy for treating patients with coronary bifurcation lesions (OR: 1.89, 95% CI: 0.60–5.95, p = 0.28), without significant heterogeneity among the studies (p = 0.52, I^2^ = 0%).

### Stent thrombosis

**Fig 3:**
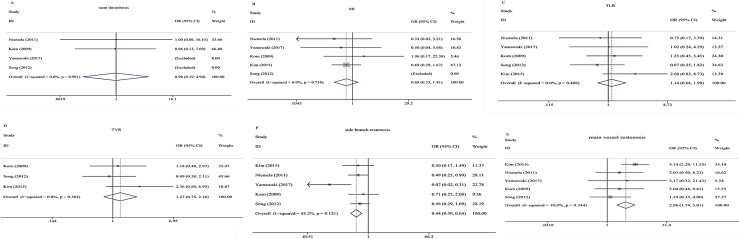
**Forest plot of secondary end points; (A) stent thrombosis; (B) MI; (C) TLR; (D) TVR; (E) main vessel restenosis; (F) side branch restenosis.** MI = myocardial infarction; TLR = target lesion revascularization; TVR = target vessel revascularization.

After pooling the data of the four studies that assessed the rate of the stent thrombosis, the risk of thrombosis was no different in patients treated with the KBI strategy compared with those received the No-KBI strategy (OR: 0.98, 95% CI: 0.19–4.94, p = 0.98; **Fig 3A**). There was no significant heterogeneity among the studies (p = 0.98, I^2^ = 0%).

### Follow-up MI

The rate of MI between the two strategies was similar (OR: 0.68, 95% CI: 0.33–1.41, p = 0.30; **Fig 3B**). No significant heterogeneity was observed (I^2^ = 0%, p = 0.76).

### TLR and TVR

Data on TVR were available in three studies. The risk of TLR and TVR was not significantly different in the KBI strategy group compared to the No-KBI strategy group [OR 1.14, 95% CI (0.68–1.90), p = 0.62; **Fig 3C** and [OR 1.27, 95% CI (0.75–2.16), p = 0.38; **Fig 3D**, respectively]. Heterogeneity was not found for TLR (I^2^ = 0%, p = 0.49) or TVR (I^2^ = 0%, p = 0.38).

### Main vessel and side branch restenosis

There was significant lower incidence of main vessel restenosis in the No-KBI strategy (OR: 2.96, 95% CI: 1.74–5.01, p<0.001); **Fig 3E**). No heterogeneity (I^2^ = 10.8%, p = 0.34) was observed. However, the No-KBI strategy was associated with a high incidence of side branch restenosis (OR: 0.44, 95% CI: 0.30–0.64, p<0.001; **Fig 3F**). The between-study heterogeneity was not significant (I^2^ = 45.2%, p = 0.12).

## Sensitivity analysis

**Fig 4:**
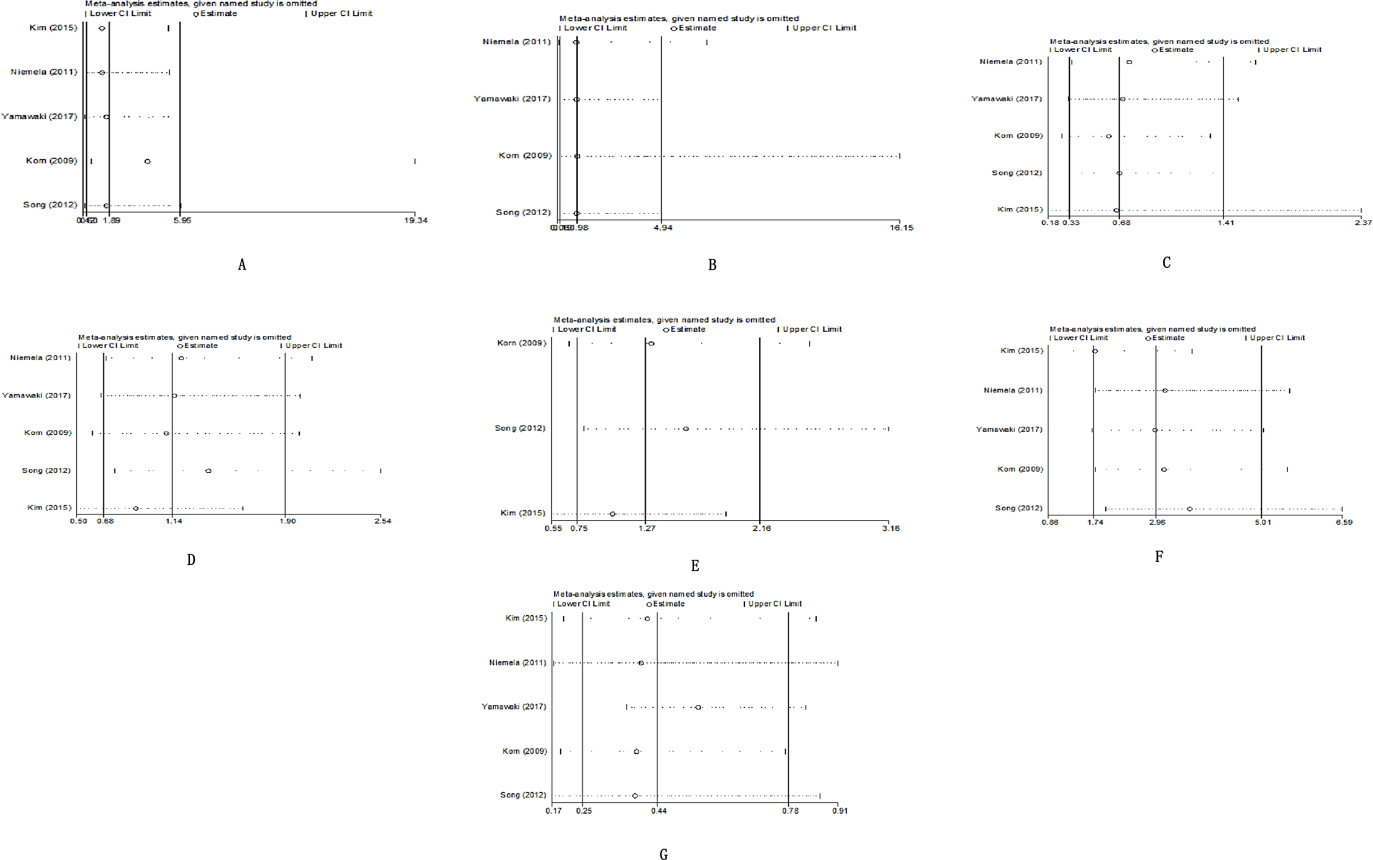
**Influence analysis for cardiac death; (A) stent thrombosis (B); (C) MI; (D) TLR; (E) TVR; (F) main vessel restenosis; (G) side branch restenosis.** MI = myocardial infarction; TLR = target lesion revascularization; TVR = target vessel revascularization.

Influence analysis demonstrated that the omission of each trial did not affect the results of whole studies (**Fig 4**).

## Publication bias

**Fig 5:**
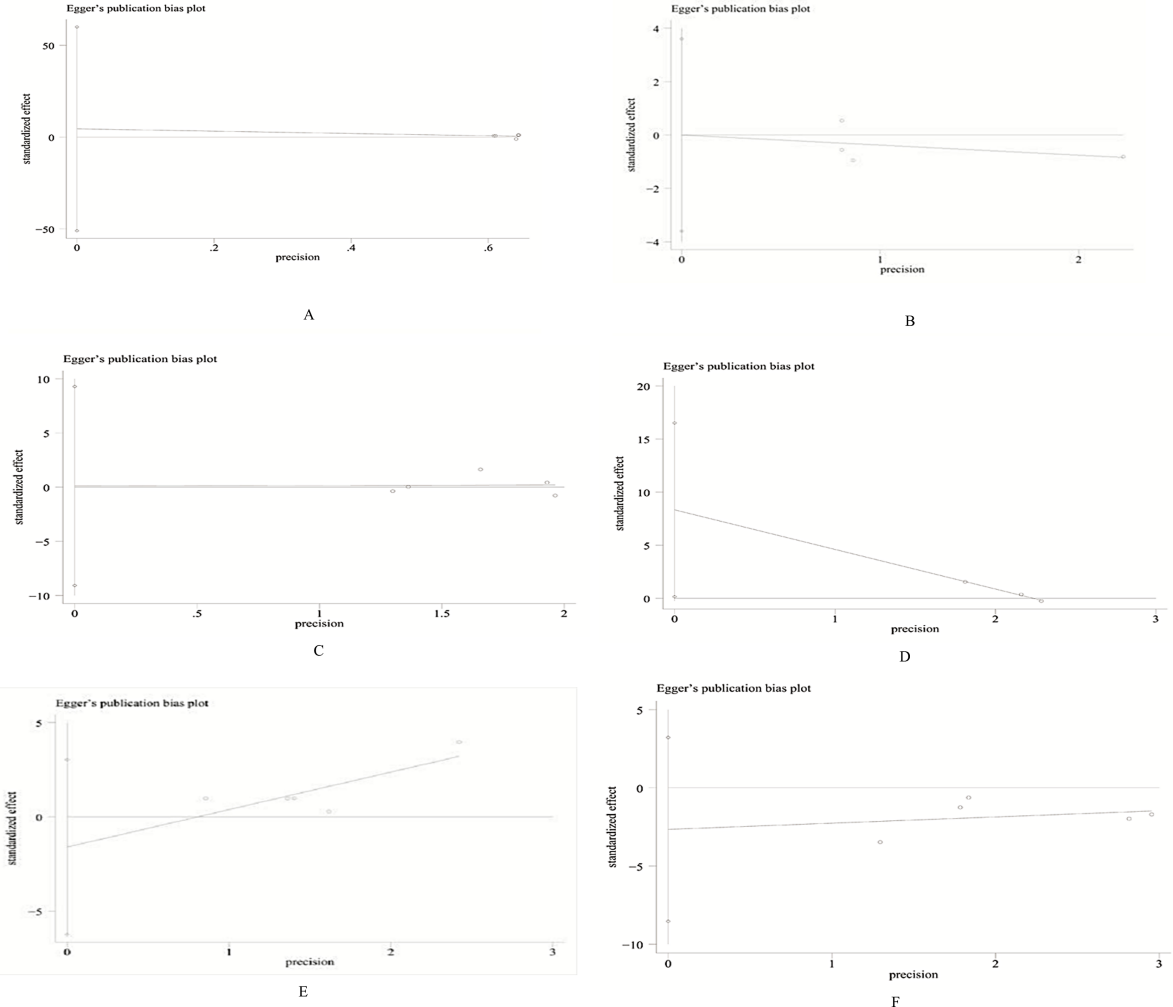
**Egger's test for cardiac death (A); MI (B); TLR (C); TVR (D); main vessel restenosis (E); side branch restenosis (F).** MI = myocardial infarction; TLR = target lesion revascularization; TVR = target vessel revascularization.

Except for TVR (Egger’s test: p = 0.049; **Fig 5D)**,There was no evidence of publication bias for cardiac death (Egger’s test: p = 0.81; **Fig 5A**), MI (Egger’s test: p = 0.99; **Fig 5B**), TLR(Egger’s test: p = 0.97; **Fig 5C**), main vessel restenosis(Egger’s test: p = 0.35; **Fig 5E**) and side branch restenosis (Egger’s test: p = 0.25; **Fig 5F**).

## Meta-regression analyses

**Fig 6:**
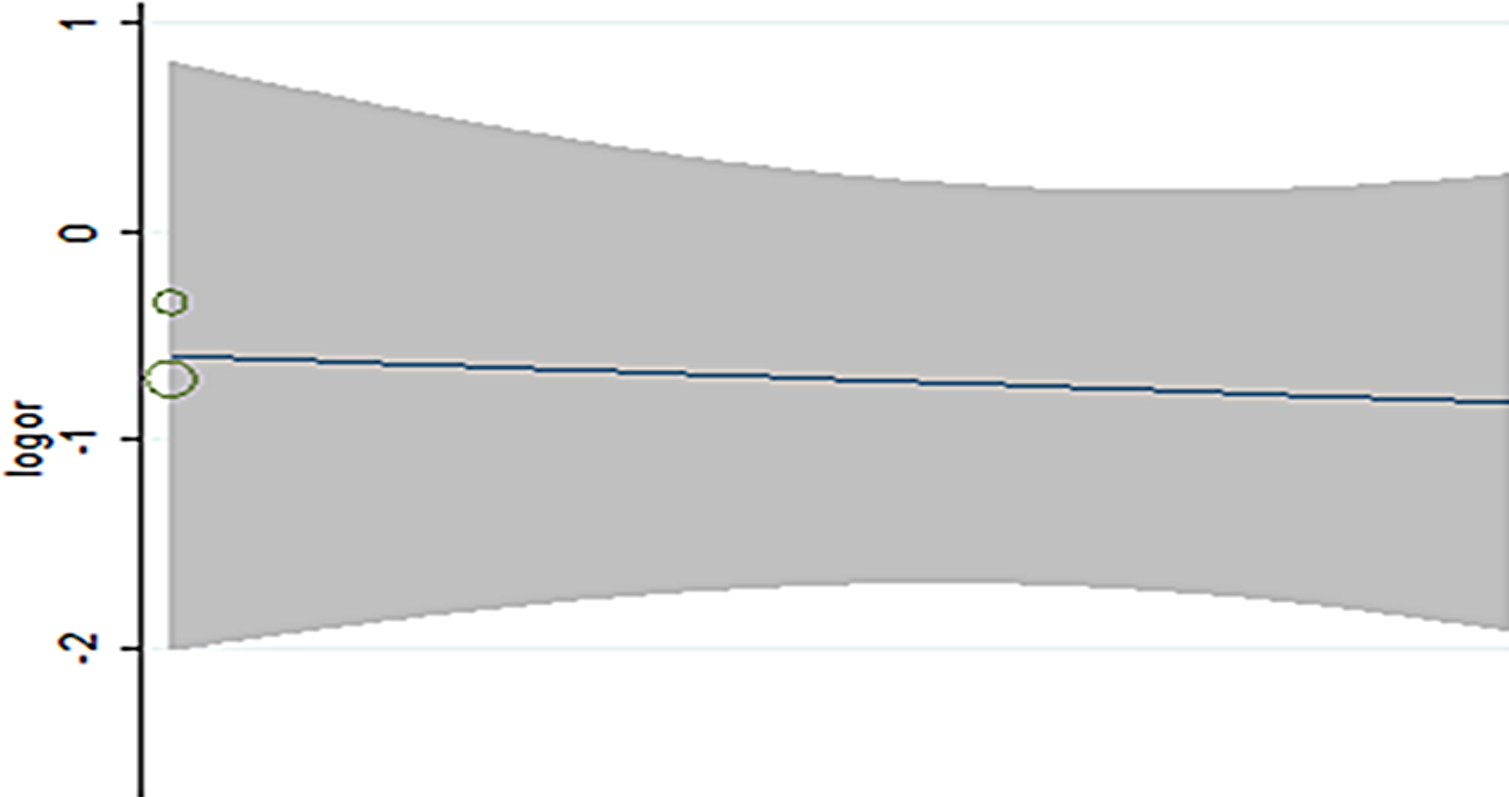
Meta-regression analyses for side branch restenosis.

As the use of IVUS and angiographic follow up which may impact side branch restenosis, Meta-regression was performed to indentify heterogeneity. But no statistical significance was observed (p = 0.54; **Fig 6**).

## Discussion

This meta-analysis compared the KBI strategy and the No-KBI strategy of treating coronary bifurcation lesions in patients undergoing PCI across 5 RCTs that included 1264 patients and showed no overall difference in clinical outcomes. Although KBI increased the risk of main vessel restenosis, it reduced the risk of side branch restenosis compared with No-KBI.

Finally, although kissing balloon inflation has been thought to be effective after main vessel stenting in order to secure side branch patency [3], recent studies have failed to show its advantages over the No-KBI strategy regarding the rate of major adverse cardiac events[5,6]. Similar to our analysis, the recently published patient-level pooled analysis of the COBIS II[7] and TAXUSPMS[8] studies reported no difference in cardiac death, MI and stent thrombosis for 3 years during follow-up. However, there was some controversy regarding whether KBI reduced the risk of TLR. Two reports indicated that the No-KBI strategy was associated with a lower risk of TLR compared with KBI[5,15]. In contrast, the COBIS II study showed that rates of TLR were higher in the No-KBI group than in the KBI[7], while our findings did not identify different rates of TLR in the two groups. However, as the small sample size does not allow for a safe conclusion to be drawn, more evidence is needed to shed light on this ongoing debate.

Interestingly, the finding of our meta-analysis is that the KBI strategy tended to increase the risk of main branch restenosis compared to the No-KBI strategy. Multiple factors could be involved. To begin with, main vessel restenosis was higher in the KBI group due to the potential elliptical deformation of the main vessel stent strut[7]. Balloons that overlap during KBI can cause the oversizing of the proximal stent segment and may lead to increased risk of main branch restenosis[16]. Then, the differential of the diameter between the proximal and distal sites may induce an increased incidence of strut malposition in the main vessel [17,18]. Furthermore, kissing balloon-induced vessel dissection and injury at the proximal edge of the implanted stent may be associated with main branch restenosis[8]. In addition, over-dilatation of the main vessel proximal segment associated with KBI would cause abnormal local hemodynamic conditions. A recent computational fluid dynamics analysis indicated that there was no benefit to side branch intervention[19]. Finally, decreased main vessel stent area associated with side branch intervention may translate into increased restenosis in the main vessel. In an intravascular ultrasound study, de Lezo js et al. reported that the distortion in the lower diamond area occurred after side branch dilatation and was not fully restored with KBI, which did not return to its initial value[20]. Generally, in the present study, KBI strategy increased the restenosis rate in the main vessel. However, this result might not be responsible for the disadvantage in the clinical outcome. The relationship between main branch restenosis and TLR remains unclear due to the limited data currently available.

The difference in side branch restenosis between patients who underwent KBI and those who underwent No-KBI in this meta-analysis was significant, and the results favored KBI. However, this result did not yield favorable clinical outcomes either, mainly because most jailed side branches do not have physiological significance[21]. Dissected or even occluded side branches are usually clinically silent and probably do not affect long-term clinical event free survival, and the majority of side branches reappear at follow-up[22,23]. Our finding supports the discrepancy between angiography and functional ischemia in side branches, consistent with the report of Koo et al.[21].

## Limitations

The present study had some limitations. First, the number of eligible trials was relatively small, which may have limited the power to detect a publication bias. There was evidence of publication bias in one of the outcomes (TVR), and therefore, the results should be interpreted with caution. Second, the duration of follow-up was quite different among the trials. Third, with regard to the baseline clinical data, there were important significant differences in patients from the KBI and No-KBI groups (e.g., left main disease, true bifurcation lesion, and use of drug-eluting stents). Finally, all the studies included in this meta-analysis were open-label, with no blinding of the operator to the technique used. These differences may have been responsible for different clinical outcomes between patient groups and could constitute a drawback of this study.

## Conclusions

This meta-analysis indicates that KBI may be associated with an increased risk of main vessel restenosis and a decreased risk of side branch restenosis. The No-KBI strategy is not inferior to the KBI strategy with regard to short- and long-term results. Consequently, routine KBI can be avoided in bifurcation lesions after main vessel stenting was uneventfully treated with the one-stent approach.

## Supporting information

S1 PRISMA Checklist Preferred Reporting Items for Meta-Analyses (PRISMA) statement checklist. (DOC)

S1 Table Quality assessment of included study. (DOC)
