# Supplementary material for: Should kissing balloon inflation after main vessel stenting be routine in the one-stent approach? A systematic review and meta-analysis of randomized trials
Source: PLoS One. 2018 Jun 27;13(6):e0197580. doi: 10.1371/journal.pone.0197580 (PMC6021082; doi:10.1371/journal.pone.0197580)
Supplement: S1 Table — (DOC) [file pone.0197580.s002.doc]

**S1 Table. Quality assessment of included study**

| Study(ref#) | Sequence generation | Allocation concealment | Blinding | | Incomplete outcome data | Selective reporting | Other bias | Single/ Multicenter | Jadad  scores |
| --- | --- | --- | --- | --- | --- | --- | --- | --- | --- |
|  |  |  | participants and personnel | outcome assessment |  |  |  |  |  |
| Nordic III [10] | Computer generate treatment allocation sequences with permuted block sizes | automated telephone allocation service provided by an independent organization | Open label | No blinding | Adequate | No | Unclear | Multicenter | 5 |
|  |  |  |  |  |  |  |  |  |  |
| CROSS [11] | Computer generate and stratify randomization sequences | Unclear | Open label | No blinding | Adequate | No | Unclear | Multicenter | 4 |
|  |  |  |  |  |  |  |  |  |  |
|  |  |  |  |  |  |  |  |  |  |
| Yamawaki et al [12] | Randomized | Unclear | Open label | No blinding | Adequate | No | Unclear | Multicenter | 4 |
|  | assignment |  |  |  |  |  |  |  |  |
| THUEBIS [13] | Computer generate randomization sequences | Sealed envelopes | Open label | Blinding | Adequate | No | Unclear | Multicenter | 5 |
|  |  |  |  |  |  |  |  |  |  |
| SMART-STRATGEY [14] | Computer-based randomization  scheme | Unclear | Open label | No blinding | Adequate | No | Unclear | Single-center | 4 |
|  |  |  |  |  |  |  |  |  |  |
